# The impact of physical activity on cardiovascular mortality in the general population

**DOI:** 10.17179/excli2021-3818

**Published:** 2021-08-06

**Authors:** Tetsuya Takahashi, Tetsu Watanabe, Harutoshi Tamura, Satoshi Nishiyama, Hiroki Takahashi, Takanori Arimoto, Tetsuro Shishido, Kazunobu Ichikawa, Sumito Inoue, Tsuneo Konta, Yoshiyuki Ueno, Takeo Kato, Takamasa Kayama, Masafumi Watanabe

**Affiliations:** 1 Department of Cardiology, Pulmonology, and Nephrology, Yamagata University School of Medicine, Yamagata, Japan; 2 Global Center of Excellence Program Study Group, Yamagata University School of Medicine, Yamagata, Japan

**Keywords:** physical activity, cardiovascular mortality, general population

## Abstract

The beneficial effect of moderate physical activity (PA) on morbidity and mortality has been observed in the general population. However, the ideal intensity of PA for improving cardiovascular longevity in Japanese general population is uncertain. The aim of this study was to investigate the relationship between the PA and cardiovascular mortality in the general population. This longitudinal cohort study included 1,826 apparently healthy subjects who participated in a community-based health checkup. There were 31 cardiovascular deaths during 10-year follow-up. Subjects were divided into 4 groups based on the quartiles of PA (low, mild, moderate and high). Kaplan-Meier analysis and multivariate Cox proportional hazard analysis demonstrated that the most favorable cardiovascular prognosis was observed in subjects with moderate PA followed by those with mild PA. High PA as well as low PA were associated with higher cardiovascular mortality compared with mild and moderate PA. Noteworthy, in subjects with high PA, Cox hazard analysis revealed that previous cardiovascular disease, smoking, brain natriuretic peptide levels, and Framingham risk score were associated with cardiovascular mortality. The results suggest a U-shaped association between cardiovascular mortality and PA. Mild to moderate PA was associated with favorable cardiovascular outcomes in the Japanese general population. High PA might be associated with poor cardiovascular outcomes in subjects with a history of heart disease and high coronary risk factors.

## Introduction

Lifestyle behaviors including smoking, unhealthy diet, physical inactivity, and sedentary behavior are closely related to the devel-opment of cardiovascular disease (Perk et al., 2012[24]). Physical inactivity was reported to be responsible for 6 % of worldwide death and 12.2 % of acute myocardial infarction (Goertzen et al., 2015[9]). It is well known that appropriate PA and exercise training can reduce cardiovascular mortality (Thompson et al., 2003[28]). The protective effect of PA on cardiovascular disease is similar to that of smoking cessation (Paffenbarger et al., 1993[23]). Increasing PA can reduce the risk of hypertension, hypercholesterolemia, type 2 diabetes mellitus, and metabolic syndrome, all of which are important cardiovascular risk factors (Lee et al., 2012[15]). On the other hand, a recent study demonstrated that the all-cause mortality rate of strenuous joggers group was equivalent to those with the sedentary group (Schnohr et al., 2015[25]). The other previous reports demonstrated that vigorous PA might not reduce cardiovascular risk as much as moderate PA whereas moderate PA was associated with lower risk of cardiovascular diseases (Armstrong et al., 2015[2]; Merghani et al., 2016[19]). Thus, it is controversial whether high intensity PA is harmful or not, and the ideal intensity of PA to reduce cardiovascular mortality is uncertain. The aim of the present study was to examine the relationship between PA and cardiovascular mortality in an apparently healthy general population.

## Methods

### Ethics statement and study population

The institutional ethics committee of Yamagata University School of Medicine approved the study, and all participants provided written informed consent. The procedures were performed in accordance with the Helsinki Declaration. This study was part of a community-based health study of inhabitants in the town of Takahata in Yamagata, Japan (total population 26,026). Community members, aged ≥ 40 years were invited to participate in this study. Between June 2004 and November 2007, 3520 subjects (1579 men and 1941 women) were enrolled in the study. Subjects completed a self-reported questionnaire to document their medical history, current medication use, and clinical symptoms. We enrolled 1826 subjects who completed the questionnaire concerning physical activities.

### Measurement

Hypertension was defined as systolic blood pressure (SBP) ≥ 140 mmHg, or diastolic blood pressure (DBP) ≥ 90 mmHg, or antihypertensive medication use. Diabetes mellitus (DM) was defined as fasting blood glucose (FBG) ≥ 126 mg/dL, glycosylated hemoglobin A1c (HbA1c) ≥ 6.5 %, or anti-diabetic medication use. Hyperlipidemia was defined as total cholesterol (TC) ≥ 220 mg/dL, triglyceride (TG) ≥ 150 mg/dL, or anti-hyperlipidemic medicine use. Obesity was defined as body mass index (BMI) ≥ 25 kg/m^2 ^(Examination Committee of Criteria for 'Obesity Disease' in Japan, 2002[7]). Metabolic syndrome was defined according to the modified National Cholesterol Education Program Adult Treatment Panel III (NCEP-ATP III) criteria, which require fulfilment of at least 3 of the following 5: BMI ≥ 25 kg/m^2^, elevated TG ≥ 150 mg/dL, reduced high-density lipoprotein cholesterol (HDL-C) < 40 mg/dL in men and < 50 mg/dL in women, elevated FBG ≥ 110 mg/dL or previously diagnosed DM, elevated SBP ≥ 130 mmHg, and/or DBP ≥ 85 mmHg or use of antihypertensive medication (NCEP, 2002[21]). Left ventricular hypertrophy (LVH) was diagnosed by a cardiologist according to the Minnesota code (1982 revised edition). Previous cardiovascular diseases were determined by self-reported questionnaires. Framingham risk score was calculated to evaluate cardiovascular risk (D'Agostino et al., 2008[3]).

### Biochemical markers

Blood samples were obtained for brain natriuretic peptide (BNP). These samples were transferred to chilled tubes containing 4.5 mg ethylene diamine tetra acetic acid disodium salt and aprotinin (500 U/mL), and centrifuged at 1,000 *g* for 15 minutes at 4 °C. The clarified plasma samples were frozen, stored at -70 °C, and thawed just before the assay was performed. BNP concentrations were measured using a commercially available radioimmunoassay specific for human BNP (Shiono RIA BNP assay kit, Shionogi Co. Ltd., Tokyo, Japan). Estimated glomerular filtration rate (eGFR) was calculated using the modification of diet in renal disease equation with Japanese coefficient (Matsuo et al., 2009[17]). Chronic kidney disease (CKD) was defined as reduced eGFR (< 60 mL/min/1.73m^2^) according to Kidney Disease Outcomes Quality Initiative clinical guidelines (Eckardt et al., 2009[4]). Insulin resistance was defined as elevated homeostasis model assessment ratio (> 2.5). Homeostasis model assessment ratio (HOMA-R) was calculated using the following equation: (FBG × fasting insulin)/405 (Matthews et al., 1985[18]). HbA1c, FBG, TC, TG, low-density lipoprotein cholesterol (LDL-C), HDL-C, hemoglobin (Hb), and creatinine were measured using standard methods.

### Assessments of PA

We assessed PA according to the Japan Arteriosclerosis Longitudinal Study Physical Activity Questionnaire (JALSPAQ) (JALS, 2008[12]). PA was quantified in terms of metabolic equivalents (METs) (metabolic equivalent (METs)・hour/day) (Hagiwara et al., 2008[10]; Ishikawa-Takata et al., 2011[11]). The JALSPAQ evaluates PA from 14 questions about occupational work, locomotion, housework, sleep time, and leisure-time activities. Occupational work includes the duration of sitting, standing, walking, and heavy work. Heavy work was defined as lifting more than 10 kg or manual labor of similar intensity. Housework PA was evaluated by duration and frequency of preparation and cleaning up of meal, laundering, room cleaning, and taking care of family members. Leisure-time PA was evaluated by duration, frequency, and type. Finally, those questionnaire data ware converted to METs・h/day (Ainsworth et al., 2000[1]). In the present study, we excluded sleep time PA to reflect true day-time PA. We divided subjects into 4 groups according to the quartiles of PA: low (< 24.4 METs・h/day, n=458), mild (24.4-28.0 METs・h/day, n=461), moderate (28.0-31.9 MTEs・h/day, n=453), and high (≧ 31.9 MTEs・h/day, n=454).

### Endpoints and follow-up

All subjects were prospectively followed for a median duration of 3391 days (interquartile range 3077-3440 days). The endpoint was cardiovascular death. Cardiovascular death was defined as death due to coronary artery disease, heart failure, arrhythmia, stroke, or aortic artery disease. The cause of death was determined by reviewing death certificates through the end of 2014. The death code (International Classification of Diseases, 10^th^ Revision) and the data on place of death were reviewed.

### Statistical analysis

Continuous data are expressed as means ± standard deviation (SD) and skewed data are presented as medians with interquartile range. Unpaired Student's *t*-tests and chi-square tests were used for comparisons of continuous and categorical variables, respectively. When the data were not normally distributed, the Mann-Whitney *U*-test was used. Differences among 4 groups based on PA were assessed using analysis of variance (ANOVA) with Tukey-Kramer Honest Significant Difference test for parametric variables, or Steel-Dwass test for nonparametric variables. Univariate and multivariate analyses with Cox proportional hazard regression were used to determine significant predictors of cardiovascular deaths. Cardiovascular risk factors including age, sex, and Framingham risk score, which were significantly associated with cardiovascular mortality in univariate Cox proportional hazard analysis, were entered into the multivariate analysis for cardiovascular mortality. We used log BNP for Cox proportional hazard analysis because BNP was not normally distributed. Cumulative overall and event-free survival rates were computed using the Kaplan-Meier method and were compared using the log-rank test. A P value < 0.05 was considered statistically significant. All statistical analyses were performed using JMP version 11 (SAS Institute Inc., Cary, NC, USA). 

## Results

### Characteristics of the study subjects

Subjects with lower PA were older than those with higher activities (Table 1(Tab. 1)). Subjects with low and high PA were more likely to be male as compared with other groups. Subjects with low PA had higher prevalence of smoking as compared with other groups. The prevalence of hypertension, CKD, and insulin re-sistance were increased with decreasing PA. SBP, DBP, HbA1c, Hb, BNP, and Framingham risk score were increased and eGFR was decreased with decreasing PA. There were no significant differences in FBG, or prevalence rate of obesity, previous cardiovascular disease, previous cancer, DM, hyperlipidemia, metabolic syndrome, LVH, and atrial fibrillation (AF) among the 4 groups.

### Cardiovascular mortality and PA

**Figure 1:**
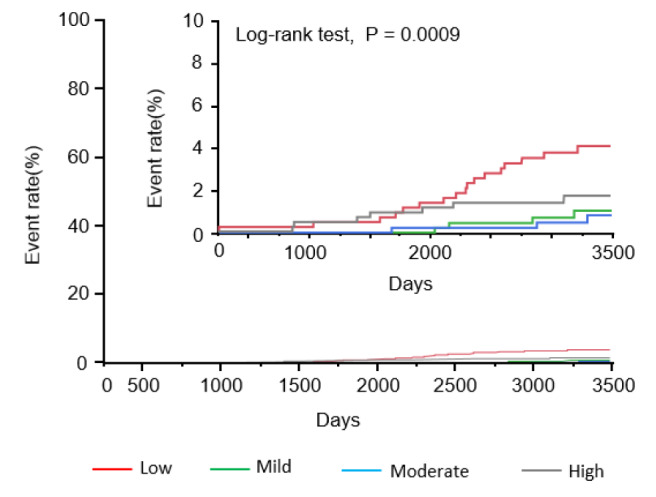
Kaplan-Meier analysis to predict cardiovascular mortality in subjects according to physical activity

During the follow-up period, there were 31 cardiovascular deaths. There were 17 cardiovascular deaths in subjects with low PA (3.7 %), 4 cardiovascular deaths in those with mild PA (0.9 %), 3 cardiovascular deaths in those with moderate PA (0.7 %), and 7 cardiovascular deaths in those with high PA (1.5 %). Kaplan-Meier analysis demonstrated that cardiovascular mortality was the highest in subjects with low PA, followed by the high PA group, and the lowest in the mild and moderate PA groups (Figure 1(Fig. 1)).

**Figure 2:**
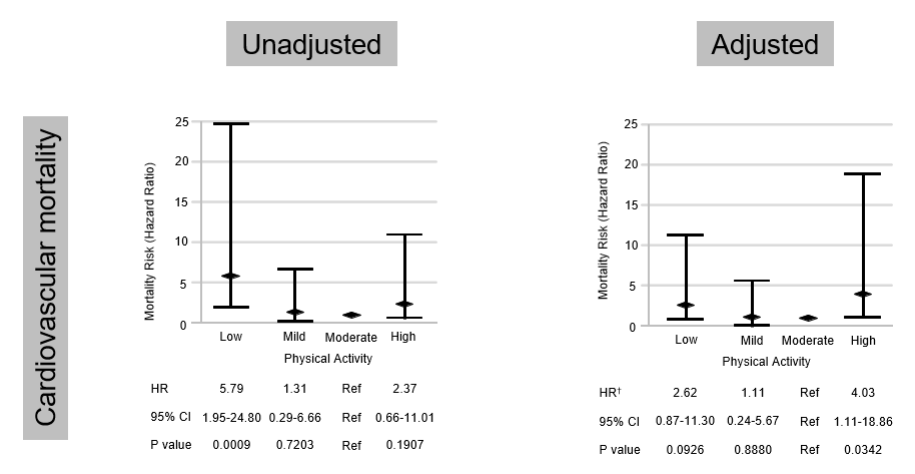
Univariate and multivariate Cox proportional hazard regression analyses for cardiovascular mortality. ^†^after adjustment for age, sex, and Framingham risk score

To determine the risk factors for cardiovascular deaths, we performed univariate and multivariate Cox proportional hazard regression analyses. In the univariate Cox proportional hazard analysis, low PA was associated with the highest mortality for cardiovascular deaths (hazard ratio, 5.79; 95 % confidence interval, 1.95-24.80; P = 0.0009), whereas high PA was associated with the second highest cardiovascular mortality but this was not statistically significant. In multivariate Cox proportional hazard analysis, high PA was associated with the highest cardiovascular mortality after adjustment for age, sex, and Framingham risk score among the 4 groups (hazard ratio, 4.03; 95 % confidence interval, 1.11-18.86; P = 0.0342) (Figure 2(Fig. 2)).

### Risk factors for cardiovascular mortality in subjects with low and high PA

We evaluated the clinical characteristic of subjects with low and high PA with cardiovascular deaths. In subjects with low PA, subjects who died during the course of the study were older and had more males, higher prevalence rates of HT, and CKD than those who survived. The prevalence rate of HL was lower in subjects with cardiovascular deaths than in those without. Subjects with cardiovascular deaths had higher SBP, BNP, and Framingham risk score than those without. eGFR was lower in subjects with cardiovascular deaths than in those without (Table 2(Tab. 2)). On the other hand, in subjects with high PA, subjects with cardiovascular deaths were older and had more males than those without. The prevalence rates of previous cardiovascular disease and smoking were higher in subjects with cardiovascular deaths than those without. Subjects with cardiovascular deaths had higher BNP and Framingham risk score than those without. eGFR was lower in subjects with cardiovascular deaths than in those without (Table 3(Tab. 3)).

To determine the risk factors for cardiovascular mortality in subjects with low and high PA, we performed univariate Cox proportional hazard regression analysis. In subjects with low PA, age, male, HT, CKD, BNP, and Framingham risk score were significantly associated with cardiovascular mortality. On the other hand, age, male, previous cardiovascular disease, smoking, BNP, and Framingham risk score were significantly associated with cardiovascular mortality in subjects with high PA. Obesity, previous cardiovascular disease, smoking, diabetes mellitus, metabolic syndrome, insulin resistance, LVH, AF, and Hb was not significantly associated with cardiovascular mortality in subjects with low PA. On the other hand, in subjects with high PA, obesity, hypertension, hyperlipidemia, CKD, LVH, AF, and Hb was not significantly associated with cardiovascular mortality (Table 4(Tab. 4)). We excluded subjects with previous cardiovascular disease, smoking, elevated BNP (≥ 40 pg/ml), and elevated Framingham risk score (≥ 10), and evaluated the impact of PA on cardiovascular mortality in remaining 295 subjects. There was no cardiovascular event in 295 subjects without previous cardiovascular disease, smoking, elevated BNP (≥ 40 pg/ml), and elevated Framingham risk score (≥ 10) (data were not shown).

Further, we evaluated the clinical characteristic of subjects with or without previous cardiovascular disease. Subjects with previous cardiovascular disease were older and had more males, higher prevalence rates of smoking than those without. Subjects with previous cardiovascular disease had higher BNP and Framingham risk score than those without. The prevalence of quartiles of PA was not significantly different between subjects with or without previous cardiovascular disease (Supplementary Table 1). 

See also the supplementary data. 

## Discussion

In this longitudinal cohort study, we showed a U-shaped relationship between cardiovascular mortality and PA. Mild to moderate PA was associated with favorable cardiovascular prognosis in the general population. Low PA was associated with higher cardiovascular mortality in the general population. Further, high PA might be associated with poor cardiovascular outcomes in subjects with a history of heart disease and high coronary risk factors.

Traditionally, regular exercise training and appropriate physical activity are well known to be associated with lower cardiovascular mortality (Fletcher et al., 1996[8]; Thompson et al., 2003[28]). Furthermore, several previous studies showed that regular exercise lowered blood pressure, body weight, triglycerides, and inflammatory biomarkers, increased high-density lipoprotein cholesterol, improved glucose metabolism and insulin sensitivity (Mann et al., 2014[16]; Szostak and Laurant, 2011[26]; Thomas et al., 2006[27]; Whelton et al., 2002[30]). Consistent with these previous studies, subjects with moderate physical activity showed the lowest cardiovascular mortality in the present study. Our results supported the fact that mild to moderate exercise are recommended to prevent future cardiovascular events.

However, recent studies suggested that high intensity PA did not reduce all-cause and cardiovascular mortality as compared with mild to moderate PA in the general population (Armstrong et al., 2015[2]; Schnohr et al., 2015[25]). In addition, several studies demonstrated that high PA was associated with increased cardiovascular mortality in subjects with cardiovascular diseases (Mons et al., 2014[20]; Williams and Thompson, 2014[31]). Although the detailed mechanism by which high PA may increase cardiovascular mortality remains unclear, long term high intensity exercise was reported to induce pathological heart remodeling, coronary artery calcification, and large artery wall stiffening (Merghani et al., 2016[19]; O'Keefe et al., 2012[22]; Schnohr et al., 2015[25]). These studies suggested that high PA might be associated with higher cardiovascular mortality in addition to low PA. On the other hand, the PURE study recently demonstrated that higher intensity PA was associated with lower cardiovascular disease risk in large subjects with different economic levels (Lear et al., 2017[14]). A Japanese cohort study also showed the same beneficial effect of high intensity PA on mortality (Kikuchi et al., 2018[13]). The effect of excessive volumes of exercise for cardiovascular mortality still remains to be controversial (Eijsvogels et al., 2016[5]). In the present study, high PA was associated with higher cardiovascular mortality as well as low PA. Our results supported the hypothesis that high intensity PA might be associated with poor cardiovascular outcomes in the Japanese general population.

Thus, high intensity PA is associated with poor cardiovascular outcomes for some individuals in the general population. However, the profile about the subjects, who are intolerant to high intensity PA and related to poor cardiovascular outcomes, remains to be determined. In the present study, subjects in high PA with cardiovascular deaths showed higher prevalence rate of previous cardiovascular disease, smoking, BNP and Framingham risk score as compared with those without although the prevalence rate of previous cardiovascular disease, smoking, BNP and Framingham risk score gradually decreased with increasing PA in all subjects. Furthermore, the prevalence rate of previous cardiovascular disease, smoking, BNP and Framingham risk score in subjects with cardiovascular deaths in high PA were higher than those in total subjects with low PA. Cox proportional hazard analysis demonstrated that the presence of previous cardiovascular disease, smoking, BNP, and Framingham risk score were significantly independent risk factors, in addition to age and sex, for cardiovascular mortality in subjects with high PA. There were several reports about the harmful effect of high PA levels in subjects who had cardiovascular disease at baseline. A previous report from Britain patients with coronary heart disease also revealed that lightly and moderately active patients showed lower cardiovascular disease related mortality as compared with inactive patients whereas vigorously active patients did not (Wannamethee et al., 2000[29]). As for heart failure with reduced ejection fraction, high intensity interval training was not superior to moderate continuous training in changing left ventricular remodeling or aerobic capacity. Moderate continuous training was said to be remained the standard exercise modality for patients with chronic heart failure (Ellingsen et al., 2017[6]). Taking these previous studies, the prevalence of a history of heart disease and high coronary risk factors may be the reason of the absence of exercise-induced health benefits for some individuals. Consistent with those results, the present study revealed that subjects with previous cardiovascular disease, smoking, high BNP levels, or high Framingham risk score might be at high risk for cardiovascular mortality even though those subjects try to exercise hard for their health benefit. On the other hand, there were no cardiovascular events in subjects without previous cardiovascular disease, smoking, high BNP levels, or high Framingham risk score. This point indicates that high intensity PA could lead to favorable cardiovascular outcomes in subjects without those risk factors.

The present study was strengthened by its inclusion of a large study population and a long follow-up period. However, there were some limitations. First, the assessment of physical activity was based on JALSPAQ, which is only used in Japan. The assessment of PA was not objective since data were collected with self-reported questionnaires. The information about PA was only obtained at baseline. Repeated assessment of PA may provide more useful information. Second, the number of cardiovascular deaths was small and the statistical power was weak for predicting cardiovascular mortality, which may affect our results. Further studies are needed to clarify this limitation.

## Conclusions

In the present study, we demonstrated the impact of PA on cardiovascular mortality in the general population. Mild to Moderate PA was associated with lower cardiovascular mortality. On the other hand, high PA might be associated with higher cardiovascular mortality as well as low PA. Especially, the presence of previous cardiovascular disease, smoking, high BNP levels might be risk factors for cardiovascular mortality in subjects with high PA in the Japanese general population.

## Acknowledgements

This study was supported in part by a Grant-in-Aid from the 21st Century of Excellence (COE) and Global COE program of the Japan Society for the Promotion of Science (15K09240). The authors thank editage (https://www.editage.jp/) for the English language review.

## Disclosures

The authors report no relationship that could be construed as a conflict of interest.

## Supplementary Material

Supplementary information

Supplementary data

## Figures and Tables

**Table 1:**
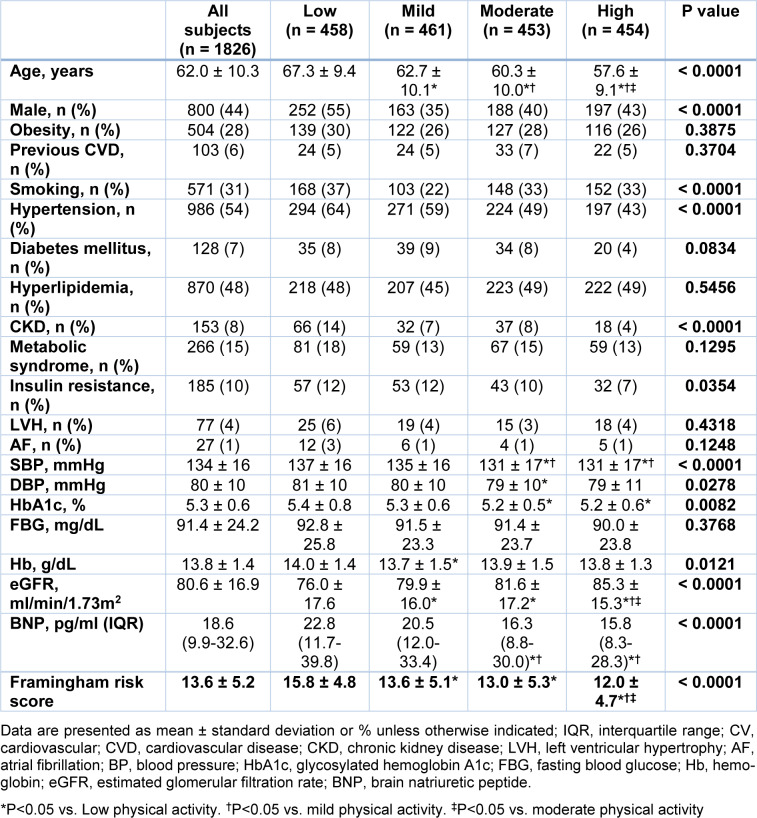
Clinical characteristics according to physical activity

**Table 2:**
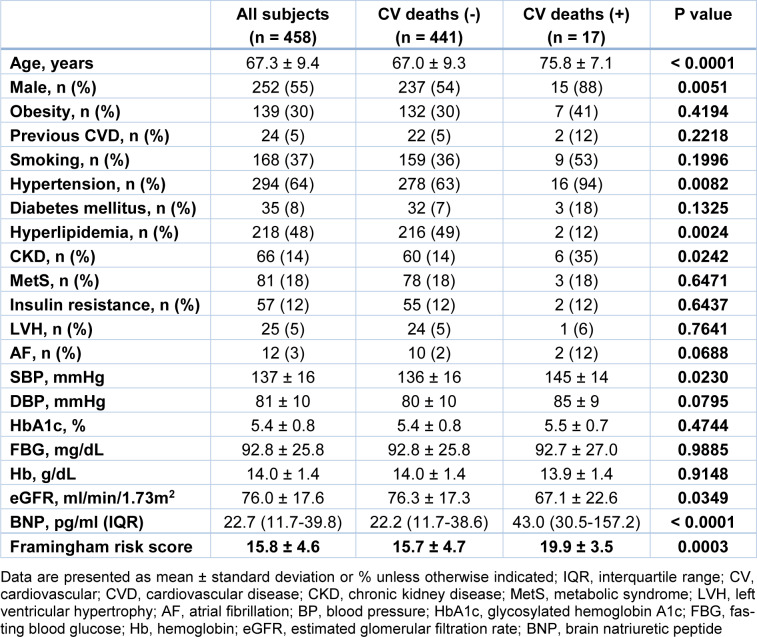
Clinical characteristics of subjects with low physical activity

**Table 3:**
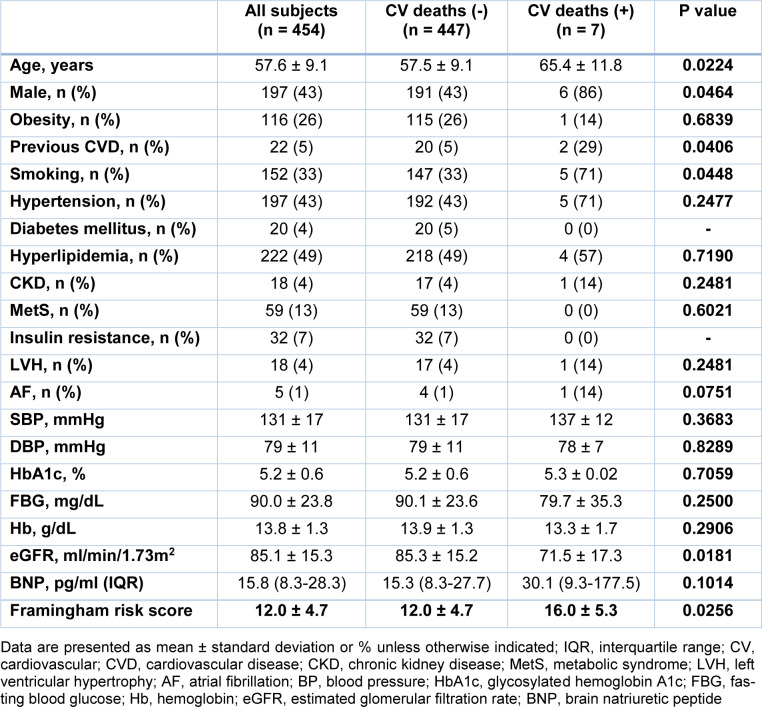
Clinical characteristics of subjects with high physical activity

**Table 4:**
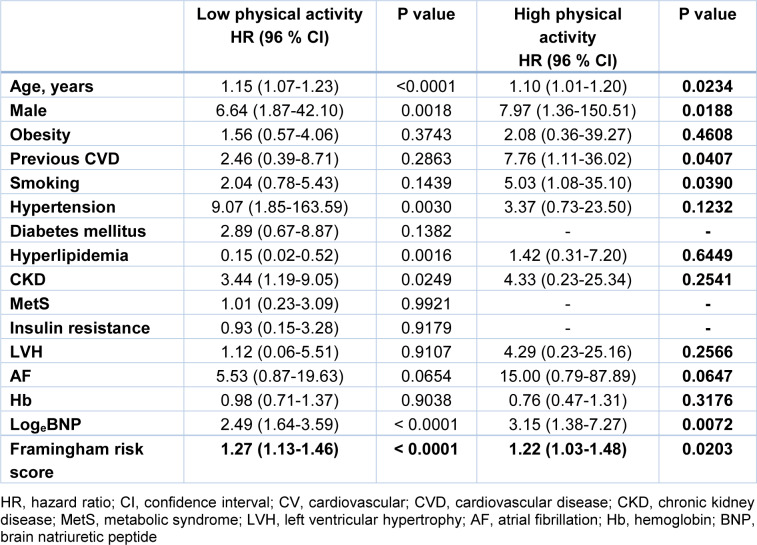
Univariate Cox hazard analysis for cardiovascular deaths
