# Supplementary material for: The impact of physical activity on cardiovascular mortality in the general population
Source: EXCLI J. 2021 Aug 6;20:1294–304. doi: 10.17179/excli2021-3818 (PMC8481793; doi:10.17179/excli2021-3818)
Supplement: Supplementary information [file EXCLI-20-1294-s-001.pdf]

EXCLI Journal 2021;20:1294-1304 – ISSN 1611-2156
Received: April 27, 2021, accepted: July 26, 2021, published: August 06, 2021

Supplementary information to:

THE IMPACT OF PHYSICAL ACTIVITY ON CARDIOVASCULAR MORTALITY IN THE GENERAL POPULATION

Tetsuya Takahashi, MD[1], Tetsu Watanabe, MD, PhD[1], Harutoshi Tamura, MD, PhD[1], Satoshi Nishiyama, MD, PhD[1], Hiroki Takahashi, MD, PhD[1], Takanori Arimoto, MD, PhD[1], Tetsuro Shishido, MD, PhD[1], Kazunobu Ichikawa, MD, PhD[1], Sumito Inoue, MD, PhD[1], Tsuneo Konta, MD, PhD[1], Yoshiyuki Ueno, MD, PhD[2], Takeo Kato, MD, PhD[2], Takamasa Kayama, MD, PhD[2], Masafumi Watanabe, MD, PhD[1]

[1] Department of Cardiology, Pulmonology, and Nephrology, Yamagata University School of Medicine, Yamagata, Japan
[2] Global Center of Excellence Program Study Group, Yamagata University School of Medicine, Yamagata, Japan

* **Corresponding author:** Tetsu Watanabe, MD, PhD, Department of Cardiology, Pulmonology, and Nephrology, Yamagata University School of Medicine, 2-2-2 Iida-Nishi, Yamagata, 990-9585, Japan, Phone: +81-23-628-5302; Fax: +81-23-628-5305; E-mail: tewatana@med.id.yamagata-u.ac.jp

http://dx.doi.org/10.17179/excli2021-3818

This is an Open Access article distributed under the terms of the Creative Commons Attribution License (http://creativecommons.org/licenses/by/4.0/).

EXCLI Journal 2021;20:1294-1304 – ISSN 1611-2156
Received: April 27, 2021, accepted: July 26, 2021, published: August 06, 2021

Supplementary Table 1: Clinical characteristics of subjects according to previous CVD

	Previous CVD (-) (n = 1723)	Previous CVD (+) (n = 103)	P value
**Age, years**	61.8 ± 10.3	64.7 ± 9.4	0.0068
**Male, n (%)**	745 (43)	55 (53)	0.0445
**Obesity, n (%)**	481 (28)	23 (22)	0.2085
**Smoking, n (%)**	529 (31)	42 (41)	0.0360
**Hypertension, n (%)**	923 (54)	63 (61)	0.1309
**Diabetes mellitus, n (%)**	125 (7)	3 (3)	0.0610
**Hyperlipidemia, n (%)**	820 (48)	50 (49)	0.8509
**CKD, n (%)**	145 (8)	8 (8)	0.8156
**MetS, n (%)**	255 (19)	11 (11)	0.2313
**Insulin resistance, n (%)**	177 (10)	8 (8)	0.3962
**LVH, n (%)**	70 (4)	7 (7)	0.2141
**AF, n (%)**	24 (1)	3 (3)	0.2686
**SBP, mmHg**	134 ± 16	135 ± 18	0.4981
**DBP, mmHg**	80 ± 10	80 ± 10	0.5053
**HbA1c, %**	5.3 ± 0.6	5.2 ± 0.4	0.2200
**FBG, mg/dL**	91.7 ± 24.1	87.4 ± 24.4	0.0843
**Hb, g/dL**	13.8 ± 1.4	14.0 ± 1.5	0.3866
**eGFR, ml/min/1.73m^2**	80.7 ± 16.9	78.9 ± 16.8	0.2788
**BNP, pg/ml (IQR)**	18.4 (9.9-32.2)	25.7 (11.8-49.0)	0.0028
**Framingham risk score**	13.5 ± 5.2	15.0 ± 4.6	0.0046
**Physical activity (low/mild/moderate high), n (%)**	434(25)/437(25)/420(24)/432(25)	24(23)/24(23)/33(32)/22(21)	0.3932

Data are presented as mean ± standard deviation or % unless otherwise indicated; IQR, interquartile range; CV, cardiovascular; CVD, cardiovascular disease; CKD, chronic kidney disease; MetS, metabolic syndrome; LVH, left ventricular hypertrophy; AF, atrial fibrillation; BP, blood pressure; HbA1c, glycosylated hemoglobin A1c; FBG, fasting blood glucose; Hb, hemoglobin; eGFR, estimated glomerular filtration rate; BNP, brain natriuretic peptide
